# Cancer screening and breast cancer family history in Spanish-speaking Hispanic/Latina women in California

**DOI:** 10.3389/fonc.2022.940162

**Published:** 2022-10-26

**Authors:** Lizeth I. Tamayo, Fabian Perez, Angelica Perez, Miriam Hernandez, Alejandra Martinez, Xiaosong Huang, Valentina A. Zavala, Elad Ziv, Susan L. Neuhausen, Luis G. Carvajal-Carmona, Ysabel Duron, Laura Fejerman

**Affiliations:** ^1^Department of Public Health Sciences, The University of Chicago, Chicago, IL, United States; ^2^Department of Public Health Sciences, University of California, Davis, Davis, CA, United States; ^3^Vision y Compromiso, Los Angeles, CA, United States; ^4^Promoters for Better Health, Pomona, CA, United States; ^5^Department of General Internal Medicine, University of California, San Francisco, San Francisco, CA, United States; ^6^Department of Population Sciences, Beckman Research Institute of City of Hope, Duarte, CA, United States; ^7^Department of Biochemistry and Molecular Medicine, University of California, Davis, Davis, CA, United States; ^8^Comprehensive Cancer Center, University of California Davis, Sacramento, CA, United States; ^9^The Latino Cancer Institute, San Jose, CA, United States

**Keywords:** breast cancer health disparities, hereditary breast cancer, cancer education, Hispanic/Latina, cancer family history

## Abstract

**Background:**

Breast cancer is the most common cancer among women in the U.S. and the leading cause of cancer death among Hispanics/Latinas (H/L). H/L are less likely than Non-H/L White (NHW) women to be diagnosed in the early stages of this disease. Approximately 5-10% of breast cancer can be attributed to inherited genetic mutations in high penetrance genes such as *BRCA1/2*. Women with pathogenic variants in these genes have a 40-80% lifetime risk of breast cancer. Past studies have shown that genetic counseling can help women and their families make informed decisions about genetic testing and early cancer detection or risk-reduction strategies. However, H/L are 3.9-4.8 times less likely to undergo genetic testing than NHW women. We developed a program to outreach and educate the H/L community about hereditary breast cancer, targeting monolingual Spanish-speaking individuals in California. Through this program, we have assessed cancer screening behavior and identified women who might benefit from genetic counseling in a population that is usually excluded from cancer research and care.

**Materials and Methods:**

The “Tu Historia Cuenta” program is a promotores-based virtual outreach and education program including the cities of San Francisco, Sacramento, and Los Angeles. Participants responded to three surveys: a demographic survey, a breast cancer family history survey, and a feedback survey. Survey responses were described for participants and compared by area where the program took place using chi-square, Fisher exact tests, and t tests. Multinomial logistic regression models were used for multivariate analyses.

**Results and Conclusion:**

We enrolled 1042 women, 892 completed the cancer family history survey and 62 (7%) provided responses compatible with referral to genetic counseling. We identified 272 women (42.8% ages 40 to 74 years) who were due for mammograms, 250 women (24.7% ages 25 to 65 years) due for Papanicolaou test, and 189 women (71.6% ages 50+) due for colorectal cancer screening. These results highlight the need of additional support for programs that spread awareness about cancer risk and facilitate access to resources, specifically within the H/L community.

## Introduction

Breast cancer is the most common cancer among women in the United States (1, 2) and the leading cause of cancer death among Hispanics/Latinas (H/L) (3). Furthermore, H/L are less likely than Non-H/L White (NHW) women to be diagnosed in the early stages of disease and are less likely to have access to high-quality care because of factors such as lower socioeconomic status (SES), high uninsured rate (3, 4), and issues communicating with providers (5). Additionally, among women of all ages dying of breast cancer, H/L have a 164% higher risk of dying before the age of 50 years in comparison with NHW women (6).

Approximately 5-10% of breast cancer cases can be attributed to inherited genetic mutations (7). Women with pathogenic variants in high penetrance genes such as *BRCA1* and *BRCA2* have a 40-80% lifetime risk of breast cancer compared to 12% risk in the general population (8). Only about 10% of mutation carriers are aware of their mutation status (9). While awareness (10) and use (11) of genetic testing in different populations has increased over time, disparities in access to hereditary breast cancer risk assessment, genetic counseling, and genetic testing continue to exist in the United States (U.S.) (12), with awareness among H/L being particularly low (33.2%) compared to NHW women (51.9%, p<0.0001) based on data from the 2010 National Health Interview Survey (9, 10, 13). Screening for pathogenic mutations can open opportunities for cancer prevention and/or engagement in frequent cancer screening to detect it early (14). Past studies have shown that genetic counseling can help women and their families make informed decisions about genetic testing and early cancer detection or risk-reduction strategies (15, 16). Genetic counseling and testing for breast cancer survivors also is critically important as it can inform targeted treatment, risk management for second primary cancers, and targeted cascade testing for at-risk family members (17). An analysis including 64,717 women who underwent genetic screening between the years 2006-2007 demonstrated that the mutation rate of BRCA1 and BRCA2 was about the same in H/L and NHW women (18, 19); however, H/L were 3.9-4.8 times less likely to undergo genetic testing than NHW women (19). The lower use of genetic testing in H/L and other underrepresented populations compared to NHW women reduces the generalizability of genetic discoveries and leads to challenges in interpreting genetic results (20).

Lack of insurance and economic concerns often are the main barriers for obtaining a genetic risk assessment for hereditary breast and ovarian cancer, and limited English proficiency and cultural factors such as embarrassment, modesty and secrecy also reduce the rate of genetic testing (21). H/L are willing to engage and have a strong desire for counseling and screening despite barriers they experience (21–25), however, within a study of 1622 participants recruited through a state cancer registry and who reported receiving genetic testing, H/L were nearly two times less likely as NHW women to report discussing genetic testing with a health provider (26, 27). A study on H/L found positive attitudes towards genetic testing for cancer prevention, with 87% agreeing it was a good idea and 87.7% agreeing that everyone should get genetic testing for cancer prevention (28). Another study focused on low income women in California, including H/Ls, identified participants at high-risk for hereditary breast and ovarian cancer *via* a phone intervention and reported that 39% accepted and received genetic counseling during the intervention period (29).

Community health educators (*promotores*) are uniquely positioned to bridge the gap between the H/L community and the health care system (30–34). Promotores are typically from the community in which they work, speak the same language, and understand the culture’s idiosincracies (32). They are able to translate medical jargon into practical, realistic steps that can be better understood and followed by members of their communities (34). Promotores-led educational interventions are cost-effective in increasing cancer screenings in the H/L community (35–39). Interventions led by promotores significantly increase breast cancer-related knowledge among participants (37, 40).

There is currently limited work on increasing breast and ovarian cancer genetic screening among H/L (10, 13, 24, 41–43). To address this gap, the research team in partnership with The Latino Cancer Institute developed a program, “Tu Historia Cuenta” (THC), to conduct outreach and educate the H/L community, particularly targeting monolingual Spanish-speaking women (44). Materials were developed to train promotores about hereditary breast cancer as well as to facilitate the interaction between promotores and the community. In this paper, we provide a description of the demographic characteristics of the participants in the program and the results of the breast cancer family history and feedback surveys which highlights the need for further improvement in hereditary breast and ovarian cancer screening in this population.

## Methods

### Study population

Recruitment of participants started in June 2020 and was led by two promotores organizations in Southern and Northern California (45, 46). As of March 2022, 1062 H/L in California had registered for the THC education session. Of these, 1042 answered the demographic survey, 891 participants answered the breast cancer family history survey, and 525 participants answered the feedback survey. The demographic survey was provided to women after registration, before the educational session. Participants were asked to answer the cancer family history survey after the education session as to maximize their comprehension of the reason for those questions and how to respond to them. As a result, a small number of participants (N=20) registered for the education session but did not complete the demographic survey and 14% (N=151) of participants attended the education session but did not answer the family history survey.

The current report is based on all survey responses available on March 18th, 2022. The inclusion criteria for participants were 1) women 21-75 years of age, 2) Spanish-speaking or bilingual, and 3) self-identifying as H/L. Participants provided verbal informed consent. Data from all surveys were de-identified. The study was approved by the University of California, San Francisco Institutional Review Board.

### Program description

THC is a promotores-led outreach and education program with materials developed using a continuous stakeholder engagement approach as previously described (44). The one-hour educational sessions provide participants basic background knowledge on breast cancer with a particular focus on hereditary breast cancer and genetics (44). THC participants completed three surveys: 1) a demographic information and general cancer screening history (i.e., mammography screening, colorectal cancer screening, cervical cancer screening) and exposure to genetic testing (i.e., cancer risk assessment) survey, 2) a breast/ovarian cancer-specific family history survey aimed at identifying women at higher risk of hereditary breast/ovarian cancer (47) that was adapted from the Pedigree Assessment Tool (48, 49), and 3) the post-education session feedback survey which assessed the utility, quality, and compressibility of the educational session components. The family history survey was selected for its ease of administration and its previous validation in low income population including H/L which was done by comparing it to genetic counselors’ assessments (50) and to Referral screening tools (RST) (48). When researchers compared the family history survey to RST, the survey had high sensitivity (~92%), specificity (0.94%) and high AUROC (98%); additional details can be found elsewhere (51). Each ‘Yes’ response on the survey had an associated score of 2, 4, or 6 depending on the age of onset and type of cancer reported for self and family member. Participants with a scores of 6 or higher were considered to have responded in a manner consistent with a strong family history of breast/ovarian cancer.

Women identified as having strong family history based on their score in the breast cancer-family history survey received a recommendation to discuss their family history with a doctor and potentially a genetic counselor. For those without a usual source of care, we provided resources and support to facilitate access.

### Survey content

The demographic survey contained questions including city of residence, zip code, age, number of years residing in the U.S., number of people in the household, and employment status. Information regarding English-language proficiency (a. monolingual Spanish speaker, b. limited English use, c. conversational English, d. fully bilingual), medical insurance (a. no insurance, b. public insurance, c. private insurance), and educational attainment (a. no school, b. elementary school, c. middle school, d. high school, e. associate degree, f. university degree) was obtained. In addition, the demographic survey contained questions regarding genetic testing such as previous knowledge and exposure to genetic testing, and interest in genetic testing. A subset of questions targeted cancer screening behavior (i.e., breast, cervical, and colorectal cancer screening).

The family history of breast cancer survey was adapted from a previously validated survey (51) and collected the following information on the participant and their first- and second-degree relatives: breast cancer diagnoses before age 50 years, after age 50 years, and cancer in both breasts. This survey included additional questions on family history of ovarian cancer, three or more family members on the same side of the family with cancer of the breast, prostate, and/or pancreas, and male family member with breast cancer. At the end of the survey, participants were asked about their willingness to be contacted in the future to learn more about their respective cancer risk if they were identified as having a strong family history of breast cancer.

The feedback survey was given to participants at the end of the education session. This survey was anonymous and had nine questions to help understand how useful participants found the information provided and whether they felt motivated to share the information learned with family and friends and to seek additional information regarding breast cancer.

### Data analysis

Average, dispersion (standard deviation-SD) and proportion measures were used to describe the characteristics of the participants and their survey responses. We used chi-square, Fisher’s exact test, and two-sided t-tests to compare characteristics and responses between participants in the three areas of outreach: San Francisco, Sacramento, and Los Angeles County, as well as by breast cancer family history score (a. <6, b.6+) and screening status.

We used multivariate multinomial logistic regression analyses to assess the association between different demographic factors and screening behavior among THC participants. The ‘never’ screened category group was defined as reference in all regression models. All analyses were conducted in RStudio version 4.1.2 (52).

## Results

### Participants’ demographic characteristics

A total of 1042 Spanish-speaking H/L women residing in San Francisco County, Sacramento and Los Angeles provided demographic information after registering for the THC education session. The average age of participants was 43 years and ranged between 21 and 73 years (**Table 1**). Most individuals were born outside the United States (86.1%) and had lived in the US for an average of 18 years (Min: 1 year, Max: 54 years). Approximately 6.5% of participants reported no formal education, 22.6% graduated from elementary school only, 16.3% middle school, 32.1% high school, 11.9% had an associate degree and 9.1% a university degree. The program’s target population was Spanish-speaking H/L, which was reflected by the responses related to English language proficiency: 17.7% were monolingual Spanish-speakers, 30.7% had basic knowledge of English, 36.9% conversational English, and 14.2% were fully bilingual. Half of the participants (50.0%) had public health insurance, 35.8% had no insurance, and 13.4% had private insurance. The average number of individuals leaving in the participants’ household was 4.3 (SD= 2.1).

**Table 1:** Demographic characteristics of 1042 ‘Tu Historia Cuenta’ program participants in California overall and by recruitment area.

Variable, N (%) or Mean (SD)	Overall	Los Angeles	Sacramento	San Francisco	p-value
Number of participants	1042 (100)	530 (50.9)	264 (25.3)	248 (23.8)	
Age in years	43.06 (10.24)	44.68 (10.22)	42.26 (9.14)	40.47 (10.79)	<0.001
Place of birth					
Foreign-born	897 (86.1)	420 (79.2)	250 (94.7)	227 (91.5)	<0.001
US-born	101 (9.7)	73 (13.8)	13 (4.9)	15 (6.0)	
Missing	44 (4.2)	37 (7.0)	1 (0.4)	6 (2.4)	
Years in the United States	18.87 (10.15)	20.43 (9.94)	19.27 (8.25)	15.56 (11.63)	<0.001
English Language Proficiency					
Monolingual Spanish Speaker	184 (17.7)	111 (20.9)	35 (13.3)	38 (15.3)	0.015
Limited English Use	320 (30.7)	152 (28.7)	97 (36.7)	71 (28.6)	
Conversational	384 (36.9)	185 (34.9)	91 (34.5)	108 (43.5)	
Fully Bilingual	148 (14.2)	76 (14.3)	41 (15.5)	31 (12.5)	
Missing	6 (0.6)	6 (1.1)	0 (0.0)	0 (0.0)	
Health Insurance Status					
No Insurance	373 (35.8)	233 (44.0)	117 (44.3)	23 (9.3)	<0.001
Public	521 (50.0)	235 (44.3)	95 (36.0)	191 (77.0)	
Private	140 (13.4)	54 (10.2)	52 (19.7)	34 (13.7)	
Missing	8 (0.8)	8 (1.5)	0 (0.0)	0 (0.0)	
Educational Attainment					
No school	68 (6.5)	55 (10.4)	3 (1.1)	10 (4.0)	<0.001
Elementary School	235 (22.6)	134 (25.3)	75 (28.4)	26 (10.5)	
Middle School	170 (16.3)	50 (9.4)	78 (29.5)	42 (16.9)	
High School	335 (32.1)	169 (31.9)	60 (22.7)	106 (42.7)	
Associate Degree	124 (11.9)	55 (10.4)	26 (9.8)	43 (17.3)	
University	95 (9.1)	54 (10.2)	22 (8.3)	19 (7.7)	
Missing	15 (1.4)	13 (2.5)	0 (0.0)	2 (0.8)	
Number of People in Household	4.32 (2.09)	4.41 (2.33)	4.62 (1.53)	3.84 (1.98)	<0.001

### Differences in demographic characteristics between participants in Los Angeles, Sacramento, and San Francisco

Average age of participants varied between the Los Angeles County, Sacramento, and San Francisco recruitment groups, with San Francisco individuals having the lowest mean age (44.7, 42.3, and 40.5 years respectively) (**Table 1**). Participants from San Francisco had been in the US for an average of 16 years (SD=12), which was lower than the number of years reported by participants in Los Angeles County and Sacramento (20 years, SD=10, and 19 years, SD=8, respectively). Furthermore, San Francisco had the largest proportion of participants with at least conversational English language proficiency and high school education or higher (**Table 1**). In Los Angeles County and Sacramento, participants were more likely to report being uninsured (44.0%, 44.3%) compared to San Francisco (9.3%). San Francisco participants were more likely to report having public health insurance (77.0% vs. 36.0% for Sacramento and 44.3% for Los Angeles County) (**Table 1**). On average, participants in Sacramento lived in larger households (4.6 people) compared to participants in Los Angeles County (4.4 people) and San Francisco (3.8 people) (**Table 1**).

### Screening behavior and knowledge about genetic testing

Most participants expressed interest in learning about genetics (98%), and only 1.3% of the individuals stated that they were not interested in learning about genetics or how genetics could be used to prevent or detect cancer early. More than half of the participants reported that they had not heard about genetic tests before (52.2%) (**Table 2**).

**Table 2:** Screening behavior and interest in breast cancer genetics among ‘Tu Historia Cuenta’ study participants (N=1,042) overall and by recruitment area.

Interest and awareness, N (%)	Overall	Los Angeles	Sacramento	San Francisco	P-value
Number of Participants	1,042 (100)	530 (50.9)	264 (25.3)	248 (23.8)	
Interest in learning about genetics and BC					
No Interest	14 (1.3)	11 (2.1)	0 (0.0)	3 (1.2)	<0.001
Some Interest	220 (21.1)	149 (28.1)	23 (8.7)	48 (19.4)	
Very Interested	801 (76.9)	364 (68.7)	240 (90.9)	197(79.4)	
Missing	7 (0.7)	6 (1.1)	1 (0.4)	0 (0.0)	
Genetic Testing Awareness					
Yes	489 (46.9)	224 (42.3)	109 (41.3)	156 (62.9)	<0.001
No	544 (52.2)	297 (56.0)	155 (58.7)	92(37.1)	
Missing	9 (0.9)	9 (1.7)	0 (0.0)	0 (0.0)	
Cancer Screening					
Breast Cancer Screening (Ages 40 to 74)	636	356	160	120	
Up to date with mammogram (<2 years ago)	357 (56.1)	187 (52.5)	97 (60.6)	73 (60.8)	0.200
Due for mammogram (never or 2+ years ago)	272 (42.8)	162 (45.5)	63 (39.4)	47 (39.2)	
Missing	7 (1.1)	7 (2.0)	0 (0.0)	0 (0.0)	
Connected to EWC Program (of those due for mammogram)	64 (23.5)	53 (32.7)	10 (15.9)	1 (2.1)	<0.001
Cervical Cancer Screening (Ages 21 to 65)	1012	512	261	239	
Ever had a pap smear	831 (82.1)	380 (74.2)	233 (89.3)	218(91.2)	<0.001
Up to date with pap smear	742 (73.3)	332 (64.8)	207 (79.3)	203 (84.9)	0.103
Due for Pap. Test (never or 3+ years ago)	250 (24.7)	165 (32.2)	52 (19.9)	33 (13.8)	
Missing	19 (1.9)	14 (2.7)	2 (0.8)	3 (1.3)	
Colorectal Cancer Screening (Age 50+)	264	158	56	50	
Up to date with colonoscopy	62 (23.5)	33 (20.9)	14 (25.0)	15 (30.0)	0.355
Due for colonoscopy	189 (71.6)	116 (73.4)	42 (75.0)	31 (62.0)	
Missing	13 (4.9)	9 (5.7)	0 (0.0)	4 (8.0)	

Among women within the age range of mammography screening guidelines (40-74 years), 56.1% were current with their mammogram (i.e., mammogram within the last 2 years), and 42.8% of the participants were due for mammograms (i.e., never had obtained a mammogram or their last mammogram was done more than 2 years ago). Of the 163 women who had never had a mammogram, 14% were navigated into the Every Women Counts program (EWC) (53), and of the 109 women who had their mammogram more than 2 years ago, 38% were navigated into this program. It is important to note that the THC education session included information about the EWC program that was shared with all participants. Due to this, women who had not previously received mammograms may not have expressed a need for navigation assistance but still taken advantage of the EWC program.

Cervical cancer screening for women between the ages of 21 to 65 years was observed for 82.1%, with 73.3% of the participants having obtained a Papanicolaou test within the last 3 years. Among participants 50 years of age and older, 23.5% reported ever having colorectal cancer screening (**Table 2**).

### Differences in screening behavior and genetic testing knowledge between participants in Los Angeles County, Sacramento, and San Francisco

Most participants in the program expressed interest in learning about genetics and breast cancer (~98%), however, a larger proportion of participants who resided in the San Francisco area were aware of genetic testing (62.9%) compared to participants in Los Angeles County (42.3%) and Sacramento (41.3%) (**Table 2**).

A similar proportion of participants in Sacramento and San Francisco were up to date with mammography screening (60.6% and 60.8%, respectively), while a lower proportion was observed among participants in Los Angeles County (52.5%); this difference was not statistically significant (**Table 2**).

Differences between regions in cervical and colorectal cancer screenings were not statistically significant (**Table 2**). However, San Francisco had the highest proportion of participants reporting a Papanicolaou test within the last 3 years (84.9%), followed by Sacramento (79.3%) and Los Angeles County (64.8%). Similarly, 30% of participants from San Francisco who were 50 years and older obtained colorectal cancer screenings, followed by 25% of participants in Sacramento and 20.9% in Los Angeles County (**Table 2**).

### Demographic characteristics and cancer screening behavior

Participant’s age, years residing in the United States, English language proficiency level, health insurance status, educational attainment, and number of residents in the household were all associated with screening behavior (**Table 3**). In general, screening was more common among bilingual participants with health insurance and formal education. Educational attainment was strongly associated with colorectal cancer screening, with up to 52% of individuals with a university degree reporting colorectal cancer screening compared to 21% of those with only elementary education and 0% of those with no formal education (**Table 3**). Education was also associated with cervical cancer screening; the largest proportion of women reporting never having had a Papanicolaou test were those with no formal education (29%) (**Table 3**). English proficiency and insurance status were associated with breast cancer screening; the lowest proportion of current mammograms was reported by monolingual Spanish speakers (42%) and the highest among those with private health insurance (72%) (**Table 3**).

**Table 3:** Cancer Screening behavior among ‘Tu Historia Cuenta’ study participants by demographic variables.

Variable	Mammography Screening*Mean (SD) or N (%)				Cervical Cancer Screening*Mean (SD) or N (%)				Colorectal Cancer Screening*Mean (SD) or N (%)		
	Up to Date	>2 years	Never	P-value	Up to Date	>3 years	Never	P-value	Yes	No	P-value
	357 (56)	109 (17)	163 (26)		742 (73)	88 (8)	162 (16)		62 (24)	189 (72)	
**Age, years**	50.5 (7.3)	51.3 (7.7)	45.5 (6.7)	<0.001	42.2 (9.2)	43.7 (8.2)	42.2 (10.8)	0.360	57 (6)	56 (6)	0.312
**Year in United States**	23.2 (9.6)	21.0 (9.7)	19.0 (8.5)	<0.001	18.2 (8.9)	20.7 (10.0)	18.4 (11.0)	0.113	28 (10)	24 (10)	0.010
**Place of birth**											
Foreign-born*	316 (57)	95 (17)	139 (25)	0.91	652 (76%)	71 (8)	131 (15)	0.077	54 (24%)	169 (76)	0.43
US-born	28 (56)	8 (16)	14 (28)		65 (66%)	10 (10)	23 (23)		7 (35%)	13 (65)	
missing	13 (45)	6 (21)	10 (34)		25 (62%)	7 (18)	8 (20)		1 (12%)	7 (88)	
**English Language Proficiency**											
Monolingual Spanish Speaker	45 (42)	22 (21)	39 (37)	0.015	114 (68)	16 (10)	38 (23)	0.003	7 (15)	41 (85)	0.01
Limited English Use	130 (59)	43 (20)	46 (21)		235 (75)	36 (12)	41 (13)		22 (24)	68 (76)	
Conversational	132 (58)	35 (15)	59 (26)		294 (80)	22 (6)	51 (14)		18 (22)	63 (78)	
Fully Bilingual	49 (64)	8 (11)	19 (25)		97 (68)	14 (10)	32 (22)		15 (47)	17 (53)	
Missing data	1 (50)	1 (50)	0 (0)		2 (100)	0 (0)	0 (0)		0 (0.0)	0 (0.0)	
**Health Insurance**											
No Insurance	104 (47)	45 (20)	73 (33)	<0.001	240 (67)	49 (14)	68 (19)	<0.001	11 (14)	67 (86)	0.013
Public	183 (60)	47 (15)	76 (25)		381 (77)	31 (6)	84 (17)		34 (27)	92 (73)	
Private	69 (72)	14 (15)	13 (14)		117 (87)	8 (6)	10 (7)		17 (37)	29 (63)	
Missing data	1 (20)	3 (60)	1 (20)		4 (100)	0 (0)	0 (0)		0 (0)	1 (100)	
**Educational Attainment**											
No school	22 (44)	11 (22)	17 (34)	0.410	36 (64)	4 (7)	16 (29)	0.030	0 (0)	36 (100)	<0.001
Elementary School	84 (58)	19 (13)	42 (29)		167 (74)	16 (7)	43 (19)		12 (21)	45 (79)	
Middle School	51 (51)	23 (23)	26 (26)		128 (76)	19 (11)	21 (12)		4 (12)	30 (88)	
High School	117 (60)	31 (16)	47 (24)		255 (79)	27 (8)	40 (12)		19 (30)	44 (70)	
Associate Degree	45 (60)	13 (17)	17 (23)		81 (69)	16 (14)	21 (18)		12 (38)	20 (62)	
University	38 (63)	10 (17)	12 (20)		68 (73)	6 (6)	19 (20)		15 (52)	14 (48)	
Missing data	0 (0)	2 (50)	2 (50)		7 (78)	0 (0)	2 (22)		0 (0)	0 (0)	
											
**Number of People in Household**	4.08 (1.8)	3.91 (1.8)	4.6 (1.7)	0.003	4.3 (1.9)	4.4 (1.9)	4.2 (1.9)	0.703	3 (2)	4 (2)	<0.001

*The sample sizes for each screening rate is based on women who answered the survey between targeted age groups: Mammography screening 40-74 years, Cervical cancer screening 21-65 years and Colorectal cancer screening ages 50+

Multiple factors were associated with mammography screening behavior in multivariate analysis. Age, educational attainment, English fluency and having private insurance were positively associated with being up-to-date with screening (**Table 4**). Additionally, program participants from Sacramento were approximately 2-fold more likely to be current with mammography screening in adjusted models compared to those from Los Angeles County (P-value 0.008)

**Table 4:** Multivariate multinomial logistic regression model testing the association between breast cancer screening behavior and demographic factors among ‘Tu Historia Cuenta’ participants ages 40 to 74 (N=587, 42 excluded from 629 due to missing data).

Variable	OR	L95%CI	H95%CI	P-value
Never had mammography (reference)				
Mammography up to date				
Age	1.17	1.12	1.22	<0.001
Years residing in the US	0.98	0.95	1.01	0.175
Immigration status (US born vs. foreign born)	0.95	0.34	2.61	0.917
Educational Attainment (ref: no schooling)				
Elementary	2.56	1.00	6.53	0.050
Middle school	1.54	0.52	4.53	0.435
High school	3.00	1.10	8.20	0.032
Associate degree	2.07	0.66	6.48	0.211
University degree	2.28	0.68	7.62	0.182
Region of residence (ref: Los Angeles)				
Sacramento	2.16	1.23	3.81	0.008
San Francisco	1.08	0.58	2.03	0.801
Insurance Status (ref: no insurance)				
Public	1.58	0.98	2.57	0.062
Private	3.19	1.46	6.98	0.004
English Fluency (ref: monolingual)				
Basic	2.38	1.14	4.99	0.021
Conversational	2.16	1.10	4.25	0.026
English fluency	1.84	0.69	4.91	0.225
Number of people in the household	0.90	0.78	1.02	0.102
**Mammography more than 2 years ago**				
Age	1.18	1.12	1.25	<0.001
Years in the US	0.97	0.94	1.01	0.118
Immigration status	0.94	0.23	3.79	0.927
Educational Attainment (ref: no schooling)				
Elementary	1.41	0.45	4.48	0.555
Middle school	2.05	0.55	7.63	0.283
High school	2.38	0.69	8.25	0.171
Associate degree	2.43	0.60	9.84	0.214
University degree	2.13	0.48	9.49	0.319
Region of residence (ref: Los Angeles)				
Sacramento	2.33	1.14	4.75	0.020
San Francisco	0.84	0.36	1.94	0.678
Insurance Status (ref: no insurance)				
Public	0.99	0.54	1.84	0.987
Private	1.63	0.61	4.33	0.329
English Fluency (ref: monolingual Spanish)				
Basic	1.60	0.65	3.98	0.307
Conversational	1.18	0.51	2.76	0.694
English fluency	0.42	0.10	1.71	0.226
Number of people in the household	0.84	0.70	0.99	0.039

Cervical cancer screening behavior was statistically significantly different when comparing participants in Los Angeles County to those in the Northern California cities, with the latter having a higher relative risk of being up to date (P-value <0.001) (**Table 5**). Participants with private insurance were 3.7 times more likely to be up to date with cervical cancer screening compared to those without health insurance (P-value 0.001).

**Table 5:** Multivariate multinomial logistic regression model testing the association between cervical cancer screening behavior and demographic factors among ‘Tu Historia Cuenta’ participants ages 21 to 65 (N=932, 80 excluded from 1012 due to missing data).

Variable	RRR*	L95%	H95%	P-Value
Never had cervical cancer screening	Reference			
Cervical cancer screening up to date				
Age	1.00	0.98	1.03	0.929
Years residing in the US	1.01	0.99	1.04	0.314
Immigration status (US born vs. foreign born)	0.56	0.26	1.23	0.150
Educational Attainment (ref: no schooling)				
Elementary	1.37	0.64	2.93	0.424
Middle school	1.37	0.56	3.40	0.492
High school	2.03	0.88	4.70	0.099
Associate degree	1.24	0.48	3.20	0.663
University degree	1.31	0.50	3.41	0.583
Region of residence (ref: Los Angeles)				
Sacramento	2.57	1.56	4.22	<0.001
San Francisco	3.71	2.05	6.71	<0.001
Insurance Status (ref: no insurance)				
Public	1.06	0.70	1.62	0.776
Private	3.73	1.67	8.34	0.001
English Fluency (ref: monolingual)				
Basic	1.21	0.65	2.28	0.548
Conversational	1.46	0.83	2.55	0.184
English fluency	0.64	0.29	1.40	0.264
Number of people in the household	1.06	0.96	1.17	0.259
**Cervical cancer screening 3 years ago**				
Age	1.02	0.98	1.06	0.419
Years residing in the US	1.03	0.99	1.07	0.158
Immigration status (US born vs. foreign born)	0.55	0.17	1.80	0.324
Educational Attainment (ref: no schooling)				
Elementary	1.16	0.30	4.49	0.828
Middle school	2.26	0.51	9.96	0.281
High school	2.82	0.69	11.54	0.150
Associate degree	3.65	0.80	16.73	0.095
University degree	1.41	0.27	7.24	0.683
Region of residence (ref: Los Angeles)				
Sacramento	2.58	1.27	5.26	0.009
San Francisco	3.20	1.32	7.74	0.010

Colorectal cancer screening behavior was statistically significantly different when comparing education attainment, with those with a high school education or higher having 6.4 times the odds of being up to date compared to those with no schooling (P-value 0.001) (**Table 6**). In addition, living in a houseful with more people was negatively associated with being current with screening (P-value 0.042).

**Table 6:** Multivariate logistic regression model testing the association between colorectal screening behavior and demographic factors among ‘Tu Historia Cuenta’ participants ages 50+ (N=240, 24 excluded from 264 due to missing data).

Never Colonoscopy as reference	OR	L95%	H95%	P-Value
Age	1.04	0.98	1.11	0.165
Years residing in the US	1.00	0.97	1.03	0.882
Immigration status (US born vs. foreign born)	0.98	0.24	3.98	0.980
Educational Attainment (ref: no schooling)				
Less than High School	2.78	1.24	6.47	0.015
High School or more	6.40	2.21	19.37	0.001
Region of residence (ref: Los Angeles)				
Sacramento	1.24	0.52	2.93	0.621
San Francisco	0.99	0.40	2.37	0.974
Insurance Status (ref: no insurance)				
Public	1.24	0.52	2.93	0.621
Private	0.99	0.40	2.37	0.974
English Fluency (ref: monolingual)				
Basic	0.79	0.26	2.54	0.681
Conversational	1.02	0.35	3.14	0.971
English fluency	1.47	0.39	5.83	0.571
Number of people in the household	0.81	0.66	0.99	0.042

### Breast cancer family history survey results

We used a previously validated family history survey to identify women with strong breast cancer family histories (51). We obtained a preliminary score and for individuals with scores of 6 or higher, we re-contacted participants to confirm their answers to the survey. THC originally identified 178 participants with a breast cancer family history score of 6 or greater (the ‘strong breast cancer family history’ category). After confirmation by the promotores, the scores changed as follows: 62 participants maintained a strong breast cancer family history score of 6+, 43 participants moved down to the ‘limited family history’ category (scores between 2 and 4), and 73 participants moved to the ‘no family history’ category (score of 0) (**Table 7**). Reasons for moving categories included: typographical errors when providing answers, answers including distant relatives, confusion between ovarian and cervical cancers, and responses based on other cancer types not linked to breast cancer risk.

**Table 7:** Breast cancer family history score and personal history of breast cancer by post confirmation score, among individuals originally placed in the ‘Strong Family History’ category.

	Original Breast Cancer Family History Score 6+*			Original Family History Score <6
New confirmed score	No Family History (0)	Limited Family History (2-4)	Strong Family History (6+)	
	N=73	N=43	N=62	N=756
**Received Genetic Counseling prior to THC**				
Yes	0 (0.0)	0 (0.0)	7 (11.3)	0 (0.0)
No	73 (100.0)	43 (100.0)	54 (87.1)	756 (100%)
Unsure	0 (0.0)	0 (0.0)	1 (1.6)	0 (0.0)
**Breast Cancer before 50 (self)**				
Yes	0 (0.0)	0 (0.0)	8 (12.9)	7 (0.9)
No	73 (100.0)	43 (100.0)	54 (87.1)	749 (99.1)
**Breast Cancer at 50+ (self)**				
Yes	0 (0.0)	3 (7.0)	1 (1.6)	4 (0.5)
No	73 (100.0)	40 (93.0)	61 (98.4)	752 (99.5)

Among the participants with a confirmed strong family history score (6+) (N=62), 7 (11.3%) had received genetic counseling before participating in THC, 8 (12.9%) reported having been diagnosed with breast cancer before the age of 50 years, and one woman (1.6%) after the age of 50 years. Among the 43 participants originally identified as 6+ that moved to the ‘limited family history’ category, 3 (7%) reported a breast cancer diagnosis after age 50 years (**Table 7**). No participants had breast cancer diagnosed in both breasts. Among participants whose original breast cancer family history score was less than 6, 7 (0.9%) women reported being diagnosed with breast cancer before the age of 50 years, and 4 (0.5%) women after the age of 50 years. Overall, there were 23 participants (2.5%) who reported a personal history of breast cancer, 15 with a diagnosis before the age of 50 years. Follow-up and navigation into genetic counseling and testing for women with a confirmed score of 6+ is currently underway.

### Demographic characteristics by family history survey results

Place of birth and educational attainment both were associated with the breast cancer family history score, with a larger proportion of U.S.-born individuals in the 6+ category (strong breast cancer family history) (15%) compared to foreign-born individuals (6%). Furthermore, the proportion of 6+ score individuals was higher among those with a university degree (16%) compared to women with lower level of educational attainment (4-9%) (**Table 8**).

**Table 8:** Breast Cancer Family History among ‘Tu Historia Cuenta’ study participants by demographic variables.

	Family History ScoreMean (SD) or N** (%)		
Variable	Strong (6+)	None & Limited(0-4)	P-value
	60 (7)	800 (92)	
**Age, years**	45.1 (10.1)	42.7 (10.2)	0.09
**Year in United States**	20.9 (9.9)	18.6 (9.6)	0.12
**Place of birth**			
Foreign-born*	46 (6)	687 (94)	0.005
US-born	13 (15)	73 (85)	
missing	1 (2)	40 (98)	
**English Language Proficiency**			
Monolingual Spanish Speaker	9 (6)	152 (94)	0.17
Limited English Use	26 (10)	245 (90)	
Conversational	16 (5)	293 (95)	
Fully Bilingual	9 (8)	106 (92)	
Missing data	0 (0)	4 (100)	
**Health Insurance**			
No Insurance	23 (7)	303 (93)	0.17
Public	25 (6)	395 (94)	
Private	12 (11)	96 (89)	
Missing data	0 (0)	6 (100)	
**Educational Attainment**			
No school	3 (5)	55 (95)	0.019
Elementary School	13 (7)	179 (93)	
Middle School	10 (7)	130 (93)	
High School	12 (4)	263 (96)	
Associate Degree	9 (9)	93 (91)	
University	13 (16)	69 (84)	
Missing data	0 (0)	11 (100)	
**Number of People in Household**	4.2 (1.5)	4.3 (2.0)	0.226

*This includes individuals who were foreign born or moved to the US before 1 year of age.

**N=860, which is 31 less than all participants with family history information due to only 860 participants having their family history confirmed up to this time.

### Feedback survey

Of the participants, 525 (50.3%) responded to the anonymous feedback survey (**Table 9**). Most of these participants found the educational materials useful when learning about hereditary breast cancer and stated that they would share the information learned from this workshop with friends and family (97.7% and 94.9%, respectively). Additionally, individuals expressed interest in obtaining more information from their family about their cancer history (93%), and 64.4% responded that they would look for further information on the internet to learn more about breast cancer. Overall, individuals felt comfortable asking questions during the workshop and felt satisfied in the manner that their questions were answered (98.5%, 99.1%, respectively) (**Table 9**).

**Table 9:** ‘Tu Historia Cuenta’ education session feedback survey responses (N=525).

Question	N (%)
Video and discussion were useful to learn about hereditary BC	
Yes	463 (97.7)
Somewhat useful	11 (2.3)
Prior awareness about hereditary genetic risk for BC	
Yes	208 (43.9)
No	266 (56.1)
Will try to obtain more information from family members about cancer history	
Yes	442 (93.2)
No	6 (1.3)
Unsure	26 (5.5)
Will look for information on the internet to learn more about breast cancer	
Yes	302 (64.4)
No	124 (26.4)
Unsure	43 (9.2)
Will share the information learned from this workshop with friends and family	
Yes	445 (94.9)
No	5 (1.1)
Maybe	19 (4.1)
Felt comfortable asking questions during the workshop	
Yes	463 (98.5)
Somewhat comfortable	7 (1.5)
Felt satisfied in the manner questions were answered	
Yes	464 (99.1)
No	3 (0.6)
More or Less	1 (0.2)
The activities conducted during the session were:	
Fundamental	372 (79.5)
Enlightening	90 (19.2)
Unnecessary	6 (1.3)
Concepts that were still confusing after the session	
None	268 (51.0)
Cancer definition	12(4.4)
Difference between benign and malignant tumor	12 (4.4)
Difference between common and hereditary breast cancer	29 (5.5)
Difference between early and advanced stages of breast cancer	21 (4.0)
What a mutation is and how is the mutation hereditary	29 (5.5)
BRCA1/2 Genes	68 (12.9)
Increased risk of breast cancer when there is a BRCA mutation	41 (7.8)
Early detection practices and preventative measures to control BC risk	23 (4.4)

Participants were surveyed regarding which topics they found confusing. Half of the participants did not report confusing topics. Thirteen percent of participants reported that they were confused about the concept of the *BRCA*1/2 genes and 7.8% about the increased risk of breast cancer when carrying a *BRCA* mutation. Other concepts covered by the program (e.g., definition of cancer, benign disease, disease stage) were still unclear by the end of the session for 4-5.5% of participants who responded (**Table 9**).

## Discussion

Tools to screen for breast cancer are important to diagnose cases early and improve outcomes (54). Disparities in breast cancer stage at diagnosis and risk of mortality between H/L and NHW women are partly due to the economic, educational, language, cultural and health care access barriers faced by members of the H/L community (3, 4). With improvement of genetic screening tools, the H/L community is at risk of being left further behind if programs are not in place to help with access and understanding of these opportunities for prevention (18, 19).

The goal of this study was to describe the results of a hereditary breast cancer outreach and education program for Spanish-speaking H/L in California and highlight the need for additional efforts to help the community move from awareness and understanding to screening and prevention.

The THC program’s target population was Spanish-speaking H/L women in three California cities and surrounding areas (San Francisco, Sacramento, and Los Angeles), who due to their limited English proficiency, socioeconomic and health insurance status, and cultural barriers, might not have access to adequate information and resources for breast cancer prevention, particularly, for prevention of hereditary breast cancer. The demographic characteristics of the program participants were consistent with the target population and supports the crucial role of promotores in connecting with underserved communities (34, 40, 55, 56).

We limited the program to women older than 21 years of age, and the average age for all participants was 43 years, with some variation by geographic area, with San Francisco participants being younger than those in Los Angeles and Sacramento. The difference in the average age of participants at the different locations might be a reflection of the age of promotores in the different groups, since the average age of an individual’s networks is likely to be concordant with their own age. For programs working with promotores, this may be important as it helps demonstrate that promotores may recruit individuals within their social circles that resemble some of their own characteristics. Having promotores of similar age of the target population of a specific program may be important.

California’s Medicaid-managed care legislation established a two-plan model in 14 counties with the largest Medicaid population (57). Medicaid recipients in these counties can choose between a local initiative and a commercial plan, with the local initiative being the state’s effort to help traditional safety net providers compete to retain Medicaid patients. The Los Angeles Care Healthy Plan and the San Francisco Health Plan resulted from this initiative. San Francisco additionally has a program called Healthy San Francisco which provides access to comprehensive health services for uninsured workers and residents of San Francisco (57, 58). The addition of the comprehensive health care program in San Francisco likely explains why a smaller proportion of individuals were uninsured (9.3%) compared to Los Angeles and Sacramento (44.3% and 44.0%, respectively). The differences in health care access across the cities and the different screening rates observed suggest universal health care may play a role in reducing disparities in cancer screening rates. Additionally, a larger proportion of participants in San Francisco had graduated from high school and had a higher level of English proficiency. Adult immigrants living and working in places where others share their ethnic backgrounds may be less likely to be proficient in English (59). This may explain some of the differences observed between English proficiency levels as H/L make up 48.6% of the population in Los Angeles, 28.9% in Sacramento and 15.2% in San Francisco. The characteristics of the promotores in the three cities might also explain the differences in the demographics of participants, even though the promotores had similar educational and linguistic backgrounds.

A study of breast cancer screening among H/L age 40 years and older in San Diego County found that 76.2% of women had received a mammogram in the past 2 years (60), which is higher than the 56.1% of H/L in our study. This difference may be because 52% of the San Diego County participants had private health insurance and a smaller percent of participants were born outside of the U.S. (76.3%). In addition, our study was conducted during the COVID-19 pandemic which may have affected cancer screening rates (61). However, other studies have also found rates consistent with what we found. A study including Mexican-American respondents of the California Health Interview Survey (CHIS) found that among women who were uninsured or had no usual source of care and were 40 years and older, 37.8-54.6% reported a mammogram in the past 2 years (62), which is consistent with the proportion in our study population. Similarly, 73.3% of women in THC were up to date with cervical cancer screening which is within the range reported for Mexican-American women in the CHIS who were uninsured or had no usual source of care (60.0-80.9%) (62). Among the THC participants who were 50 years and older, 23.5% had obtained a colorectal cancer screening; this percentage is lower compared to past studies that identified 50.2-60% of H/L California residents aged 50 years and older who had ever received colorectal cancer screening (63, 64) but is similar to findings from a Northern California catchment area population assessment (65).

The THC participants who had never obtained a mammogram reported a higher average number of household members, which is a measure that correlates with socioeconomic status, thereby suggesting that participants who never had a mammogram within the THC study may also be those in the lowest income bracket.

Participants expressed interest in learning about hereditary breast cancer and genetics despite limited knowledge at the time of registration. The proportion of participants identified as having strong family history of breast cancer (~7%), is concordant with other estimates in studies assessing breast cancer family history in unaffected women (66–68). The larger proportion of women with a high breast cancer family history score among U.S.-born (15%) compared to foreign-born participants (6%) might be due to differences in the flow of information about cancer family history in these two groups. A similar interpretation can be posed for the higher proportion of women with university degrees with strong breast cancer family history. Comparing the rate of high penetrance mutations by place of birth and reported family history of cancer could provide important information about the carrier status predictive accuracy of the breast cancer family history survey by immigration status/generation among H/L in California.

There were 116 individuals whose breast cancer family history survey scores changed after a second conversation with promotores. Over-reporting of cancer family history has been noted in previous studies (69, 70). The most common reasons for the discordance between the original survey response and that after a second contact were unintentional errors when choosing options and confusion about type of cancer in the family (e.g., ovarian vs. cervical, which has been previously described (71)). Only participants who had initially had a high family history score (greater than or equal to 6) were part of the confirmation group, which could lead to underestimation of the proportion of participants in the strong family history category.

A strength of this study was that we were able to connect to a population that is often excluded from health studies (35.8% of the study participants did not have health insurance and ~48% were either monolingual Spanish-speakers or had limited English proficiency). Another strength was that researchers worked closely with promotores to ensure the relevance and accessibility of the study materials and process, while engaging community members to obtain their perspective and perceptions of the program (44). Due to the pandemic, all the education sessions were held virtually. Hosting sessions virtually allowed more women to participate, as usual barriers for in person education were removed (e.g., transportation, child and elderly care responsibilities).

The study has some limitations. Participants were enrolled through the work of two organizations and individuals were recruited from promotores’ social circles and networks. Consequently, results from this study may not be generalizable to the overall population of Spanish-speaking H/L in San Francisco, Sacramento, and Los Angeles County. Additionally, the education program advertised learning about hereditary breast cancer, which could have influenced people to participate if they had a personal interest based on their family history of cancer. However, the percentage of individuals in the THC study identified as candidates for genetic counseling (7%) was slightly less than what has been reported for the general population of unaffected women in the U.S. (8% to 12%) (66–68), suggesting that the study sample is not enriched for people with strong family history of breast cancer.

Overall, participants found the THC education session to be useful, and most of the participants reported willingness to share the information they acquired in the session with their friends and family. We hope the sharing of information will lead to greater awareness about hereditary breast cancer in California Spanish-speaking H/L communities.

## Conclusion

The THC promotores-led outreach, education and breast cancer family history assessment program implemented in San Francisco, Sacramento, and Los Angeles in June 2020 has reached more than 1000 Spanish-speaking H/L. Since then, we have identified 62 women (7%) which based on survey responses could benefit from genetic counseling, 272 (42.8%) women due for mammograms (64 of whom we have navigated to the EWC program), 250 (24.7%) due for Papanicolaou test, and 189 (71.6%) due for colorectal cancer screening. Follow-up of the THC participants who were referred to and/or navigated to genetic counseling and testing will be important to assess the long-term impact of the program on the prevention of advanced breast cancer diagnosis among Spanish-speaking H/L with strong family history of the disease.

The results from the THC study highlight the need for additional programs targeted to this underserved population in order to spread awareness about cancer risk and facilitate access to resources for prevention.

## Data availability statement

The raw data supporting the conclusions of this article will be made available by the authors, without undue reservation.

## Ethics statement

The studies involving human participants were reviewed and approved by University of California, Davis IRB. The patients/participants provided their verbal informed consent to participate in this study. The current study is not subject to HIPAA rules and therefore waived from written informed consent requirements.

## Author contributions

LT performed the statistical analysis and wrote the manuscript. LF conceived and designed the study and provided overall supervision of data analyses and manuscript writing. YD contributed to the conception and design of the study and to manuscript revision. FP controlled the quality of the data, organized the database, and contributed to manuscript revision. MH and AM supervised data collection. XH and VZ contributed to data curation and manuscript revision. EZ, SN, and LC-C contributed to manuscript revisions. All authors contributed to the article and approved the submitted version.

## Funding

EZ, SN, LGC-C and LF received support from the California Initiative to Advance Precision Medicine (contract OPR18111). LF receives support from the National Cancer Institute (R01CA204797). LGC-C cancer health disparities and community engagement efforts receive support from the NCI (P30CA093373, U54CA233306 and R01CA223978), National Center for Advancing Translational Science (UL1TR001860), the Heart, BrEast and BrAin HeaLth Equity Research (HEAL-HER) Program and the Auburn Community Cancer Endowed Chair in Basic Science. The opinions expressed in this article are the author’s own and do not reflect the view of the National Institutes of Health, the Department of Health and Human Services, or the United States government.

## Acknowledgments

We want to thank the ‘Tu Historia Cuenta’ participants for taking the time to attend educational sessions and respond to study surveys. In addition, this study would not have been possible without the passion, resilience and energy of the promotores who faced and overcame the difficulties of implementing a virtual outreach and education hereditary breast cancer program in the middle of the Covid19 pandemic.

## Conflict of interest

The authors declare that the research was conducted in the absence of any commercial or financial relationships that could be construed as a potential conflict of interest.

## Publisher’s note

All claims expressed in this article are solely those of the authors and do not necessarily represent those of their affiliated organizations, or those of the publisher, the editors and the reviewers. Any product that may be evaluated in this article, or claim that may be made by its manufacturer, is not guaranteed or endorsed by the publisher.
